# SV40 T antigen helicase domain regions responsible for oligomerisation regulate Okazaki fragment synthesis initiation

**DOI:** 10.1002/2211-5463.13373

**Published:** 2022-02-07

**Authors:** Nichodemus O. Onwubiko, Felicia Scheffel, Ingrid Tessmer, Heinz Peter Nasheuer

**Affiliations:** ^1^ Biochemistry School of Biological and Chemical Sciences Biomedical Sciences Building NUI Galway Galway Ireland; ^2^ Rudolf Virchow Center for Experimental Biomedicine University of Würzburg Würzburg Germany

**Keywords:** DNA polymerase α‐primase (Pol α), eukaryotic DNA replication, initiation reaction, Okazaki fragment synthesis, replication protein A (RPA), SV40 large T antigen

## Abstract

The initiation of Okazaki fragment synthesis during cellular DNA replication is a crucial step for lagging strand synthesis, which is carried out by the primase function of DNA polymerase α‐primase (Pol‐prim). Since cellular replication protein A (RPA) prevents primase from starting RNA synthesis on single‐stranded DNA (ssDNA), primase requires auxiliary factors, such as the simian virus 40 (SV40) T antigen (Tag), for the initiation reaction on RPA‐bound ssDNA. Here, we investigated the ability of Tag variants and Tag protein complexes to bind to ssDNA and their resulting effects on the stimulation of Pol‐prim on free and RPA‐bound ssDNA. Atomic force microscopy imaging showed that while Tag_131‐627_(V350E/P417D) and Tag_131‐627_(L286D/R567E) (abbreviated as M1 and M2, respectively) could bind to ssDNA as monomers, these monomeric Tags could come together and bind to ssDNA as dimers as well. In a model assay for the initiation of Okazaki fragment synthesis, full‐length Tag SV40 Tag_1‐708_ and monomeric M2 stimulated DNA synthesis of Pol‐prim on ssDNA and on RPA‐bound ssDNA. In contrast, neither monomeric M1 nor M1‐M2 dimers could stimulate Pol‐prim, on ssDNA or on RPA‐bound ssDNA. Overall, we show that a lack of stimulatory activity of monomeric M1 and M1‐M2 dimers suggests that residues V350 and P417 are not only important for interactions between Tag molecules but also for protein–protein interactions within Okazaki fragment initiation complexes. Thus, we highlight that mutations in M1 are dominant negative with regard to Okazaki fragment initiation.

## Graphical abstract

The oligomerisation of the SV40 T antigen (Tag) is required for DNA helicase activity at the replication fork. Mutations in Tag controlling its oligomerisation also modulate its interactions with DNA polymerase α‐primase (Pol‐prim) and replication protein A (RPA) during the initiation of Okazaki fragment synthesis on the lagging strand.

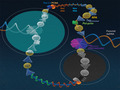

**Abbreviations**
AAA+: ATPases associated with diverse cellular activities
AFM: atomic force microscopy
bp: base pair
CMG: Cdc45‐Mcm2‐7‐GINS
CTF4/AND1/WDHD1: chromosome transmission fidelity factor 4/acidic nucleoplasmic DNA‐binding protein/WD repeat and HMG‐Box DNA binding protein
dsDNA: double‐stranded DNA
EMSA: electromobility shift assay
M1: Tag variant Tag131‐627(V350E/P417D)
M2: Tag variant Tag131‐627(L286D/R567E)
MFP: molecular force probe
MLP: molecular lipophilicity potential
NCCR: noncoding control region
nt: nucleotide
OB: oligonucleotide/oligosaccharide‐binding
OBD: origin‐binding domain
p180N: N‐terminal sequence of p180 in Pol‐prim
p68N: p68 N‐terminal region
PCNA: proliferating cell nuclear antigen
PDB: protein data bank
Pol δ: DNA polymerase δ
Pol‐prim: DNA polymerase α‐primase
Prim 1: small primase subunit p48
Prim2: large primase subunit p58
RFC: replication factor C
RPA: replication protein A
RPA32C: C‐terminal protein interaction domain of RPA subunit RPA32
ssDNA: single‐stranded DNA
SV40: simian virus 40
Tag: T antigen
wt: wild‐type

Studies of the mechanism of DNA replication and how eukaryotes duplicate their genomes accurately during the cell cycle are at the forefront of molecular biology research [1, 2, 3, 4, 5]. The replication of the genomes of small eukaryotic DNA viruses such as simian virus 40 (SV40) and other polyomaviruses has served as a model for the understanding of cellular DNA replication of their hosts [6, 7, 8]. Their DNA replication depends mainly on host factors, and only the multifunctional viral protein T antigen (Tag) is required for viral DNA replication *in vitro*. At the start of each round of viral DNA replication, Tag binds as a double hexamer to the double‐stranded DNA (dsDNA) of its origin located in the noncoding control region (NCCR) of the virus genome [7, 8]. Tag then destabilises and unwinds the dsDNA within the NCCR yielding single‐stranded DNA (ssDNA). The eukaryotic ssDNA‐binding protein, replication protein A (RPA), binds to and stabilises the ssDNA, which is the substrate for the initiation of viral replication by DNA polymerase α‐primase (Pol‐prim) [9, 10, 11, 12, 13, 14]. The primase function of the enzyme complex synthesises the first RNA primer on the leading strand and extends it via its DNA polymerase activity. This RNA‐DNA primer is then bound by proliferating cell nuclear antigen (PCNA) with the support of replication factor C (RFC) in an ATP‐dependent fashion. Finally, the primer‐PCNA complex is bound by DNA polymerase δ (Pol δ), which synthesises the leading strand in a continuous fashion [8, 15, 16].

In parallel, the lagging strand must be synthesised in a discontinuous fashion by the formation of multiple Okazaki fragments with an average length of 250–300 nucleotides (nts) [4, 8, 10]. Here, the initiation of Okazaki fragments during eukaryotic DNA replication is a crucial step for lagging strand synthesis that is also carried out by Pol‐prim. It is important to note that the primase is fully capable of synthesising primers on ssDNA as a substrate, but the presence of RPA on ssDNA prevents primase to start RNA synthesis. Thus, Pol‐prim requires auxiliary factors such as Tag for the initiation reaction on RPA‐bound ssDNA [8, 12, 17, 18, 19, 20]. The RNA‐DNA primer synthesised on the lagging strand by Pol‐prim is then again recognised by RFC, which loads PCNA onto the 3′‐end of the primer allowing Pol δ to efficiently recognise the RNA‐DNA and synthesise the Okazaki fragment in a processive manner. The RNA containing Okazaki fragments now need to be further processed and matured until DNA ligase 1 finally ligates two newly synthesised Okazaki fragments to form longer stretches of continuous DNA [2, 4, 5].

RPA plays important roles in DNA modifying processes in eukaryotes by protecting ssDNA from degradation, preventing its re‐annealing, and in recruiting various protein factors to the DNA [11, 12, 13, 14, 21]. RPA is a heterotrimeric protein complex consisting of three subunits RPA70, RPA32, and RPA14 named according to their size. RPA70 has four main domains, oligonucleotide/oligosaccharide‐binding (OB) folds F, A, B, and C from N to C terminus, whereas RPA32 and RPA14 contain OB folds D and E, respectively. The OB folds of RPA are involved in DNA and protein binding. In addition to the OB fold, RPA32 has an N‐terminal phosphorylation region and a C‐terminal protein interaction domain (RPA32C), which both are involved in the regulation of RPA functions in the eukaryotic DNA metabolism [11, 12, 13, 14, 21]. Due to its tight binding to ssDNA, multiple mechanisms have evolved in eukaryotic DNA replication, repair and recombination pathways to destabilise RPA‐ssDNA complexes [22, 23, 24]. The modular mechanism of RPA interactions with ssDNA using its multiple OB‐fold DNA binding domains and kinetics of these interactions provide gateways to destabilise RPA‐ssDNA complexes even by proteins having lower affinities to ssDNA than RPA [24]. For example, negatively charged proteins such as DSS1 interact with the large subunit RPA70, attenuate the binding of RPA to ssDNA and allow the DSS1 binding partner BRCA2 to load Rad51 onto ssDNA replacing RPA and allowing homologous DNA recombination (for more detail see Ref. [23]). Alternatively, proteins such as Rad52 and SV40 Tag are thought to bind to the C terminus of RPA32 adjacent to the OB‐fold D and destabilise the RPA‐ssDNA thus allowing the replacement of RPA by other proteins such as Rad51 or giving access to enzymes such as Pol‐prim to ssDNA (for more details, see Ref. [22, 24]).

Pol‐prim is a heterotetrameric protein complex consisting of p180, p68, p58 and p48 with the two largest subunits forming the Pol α‐core and the two smallest making up the primase with its two subunits also called Prim 1 (p48) and Prim 2 (p58) [1, 10, 25]. Since DNA polymerases lack the ability to perform *de novo* DNA synthesis without a free 3’ hydroxyl group, eukaryotic replicative DNA polymerases utilise the free OH group of an RNA primer synthesised by the primase function of Pol‐prim [26]. During DNA replication, primase synthesises RNA primers with a size of ~ 10 nts, which are then transferred to the p180 subunit of Pol‐prim adding 20–30 dNMPs to generate RNA‐DNA hybrid primers that are subsequently elongated by highly processive and high fidelity DNA polymerases. To achieve its functions, Pol‐prim also interacts with multiple proteins such as the Cdc45‐Mcm2‐7‐GINS (CMG) complex, chromosome transmission fidelity factor 4/acidic nucleoplasmic DNA‐binding protein/WD repeat and HMG‐Box DNA binding protein (CTF4/AND1/WDHD1), RPA and SV40 Tag [2, 4, 5, 27, 28].

To study the binding mechanism of Tag to dsDNA sequences, Chang et al. (2013) [29] established two double mutants, M1 (Tag_131‐627_ V350E/P417D) and M2 (Tag_131‐627_ L286D/R567E) in a shortened Tag variant wild‐type (wt) Tag_131‐627_. They showed that the M1 and M2 proteins are monomers in solution, but, after mixing them together, they associated with each other forming stable dimers [29]. Recently, we have shown that monomeric and not hexameric Tag functions as auxiliary factor for Pol‐prim during Okazaki fragment synthesis [30]. In the present study, the ability of Tag variants and Tag protein complexes to bind to ssDNA and their activity in the stimulation of Pol‐prim on free and RPA‐bound ssDNA was investigated. The monomeric Tag mutant M2 bound ssDNA as monomer, whereas mixing M1 and M2 together they bound to ssDNA as dimers but not as higher oligomeric forms. Interestingly, wt Tag_131‐627_, the M1 protein, and the M1‐M2 dimer inhibited Pol‐prim‐dependent DNA synthesis on ssDNA, whereas full‐length Tag and M2 stimulated the reaction. In the presence of RPA, Pol‐prim‐dependent DNA synthesis is inhibited. Full‐length Tag and M2 stimulated the incorporation of radioactive dNMPs by Pol‐prim on RPA‐bound ssDNA, whereas wt Tag_131‐627_ inhibited the reaction even further. M1 did not show any activity in the assay in the presence of RPA, whereas M1‐M2 dimers slightly stimulated Pol‐prim‐dependent DNA synthesis. These findings have important implications on the mechanism of Pol‐prim activation by SV40 Tag, which will be discussed.

## Materials and methods

### Protein expression and purification

SV40 Tag and the Pol‐prim complex were expressed using a baculovirus expression system and purified as described [30, 31, 32]. The purified proteins were examined by SDS/PAGE (Hoefer) followed by Coomassie Brilliant Blue staining and western blotting as described in [33]. Additionally, shortened constructs of Tag (wt Tag_131‐627_) and mutant variants thereof were expressed in *E. coli* and purified from *E. coli* cultures as described by Onwubiko et al. [30]. Human RPA was expressed in *E. coli* as heterotrimeric complex and purified as previously described [34, 35]. The purified replication proteins were analysed by SDS/PAGE followed by Coomassie Brilliant Blue staining and were at least 95% homogeneous. After verifying their purity, they were stored in small aliquots at −80 °C until later use. UV absorbance spectrophotometry using a NanoDrop 2000 Spectrophotometer (Thermo Fisher Scientific, Cork, Ireland) was used to determine protein concentrations. Absorbance spectra were collected from 250 to 300 nm and confirmed by Bradford assay (Bio‐Rad, Hercules, CA, USA).

### Protein structure analyses

Protein structure data were downloaded from the RCSB Protein Data Bank (PDB) and analysed using the chimerax program [36].

### DNA substrates

In the electromobility shift assay (EMSA) experiments, the labelled 57‐mer ssDNA oligonucleotide ssDNA(GAPDH) (5′‐CGA CAG TCA GCC GCA TCT TCT TTT GCG TCG CCA GCC GAG CCC TAT AGT GAG TCG TAT‐3′) was used [30]. The oligonucleotide was radioactively 5′‐end‐labelled using T4 polynucleotide kinase (New England Biolabs (NEB)) following the manufacture’s procedures using 30 μCi P32 γ‐ATP (3000 Ci·mmol^−1^, PerkinElmer, Dublin, Ireland). Then, GE Healthcare spin columns were used to purify the labelled oligonucleotide. For atomic force microscopy (AFM) experiments, DNA substrates were prepared as previously described [30, 37]. The gapped substrate consists of a central 48 nt ssDNA region between ~ 250  base pairs (bp) on either side (ssDNA region at 50% of the DNA fragment length).

### DNA binding assays

For EMSA, radioactively labelled ssDNA oligonucleotides (GAPDH ssDNA primer, see above) were incubated with the indicated amounts of proteins in binding buffer (10 mm HEPES‐KOH, pH 7.8, 1 mm EDTA and 40 mm NaCl) for 30 min in a volume of 15 μL) and then loaded on a native polyacrylamide gel. The free and protein‐bound DNA were separated by EMSAs as previously described [30, 38, 39]. Autoradiography of the gels was carried out with a Fuji FLA5100 phosphor‐imager (Fujifilm, Düsseldorf, Germany) and free ssDNA and oligomeric Tag‐ssDNA complexes were quantified using the program imagequant (Fujifilm).

For AFM analyses, 500 nm SV40 Tag (full‐length protein or Tag_131‐627_ wt or mutants) were incubated with the gapped DNA substrate (3–5 nm) in AFM buffer (25 mm HEPES‐NaOH, pH 7.5, 25 mm sodium acetate, 10 mm magnesium acetate, 1 mm ATP) for 1 h at room temperature (RT) as described earlier [30]. Following incubation, samples were cross‐linked with 0.1% glutaraldehyde for 10 min at RT. As negative control, the DNA substrate was also imaged in the absence of protein at a concentration of 4 nm. AFM imaging was performed using a Molecular Force Probe (MFP) 3D AFM (Asylum Research, Oxford Instruments, Santa Barbara, CA, USA) and AC240TS probes (Olympus, Tokyo, Japan). Experiments were carried out at least in duplicate. DNA lengths, the 50% position on the DNA substrates (where the ssDNA stretch is located), and volumes of protein complexes bound at this 50% position (or the ssDNA in the negative control) were determined using imagej (NIH software), Image SXM (Steve Barret, University of Liverpool) and Origin (Origin Lab Corporation, Northampton, MA, USA) as described [30].

### DNA polymerase α‐primase activity on ssDNA

The Pol‐prim assays measured primase‐initiated DNA synthesis on unprimed ΦΧ174 ssDNA and on RPA‐bound ssDNA as previously described using SV40 DNA replication conditions [19, 30, 40] with slight modifications. In short, Tag samples (0.3 and 0.6 µg of full‐length protein or shortened monomer variants as indicated) were incubated in buffer containing 30 mm HEPES‐KOH, pH 7.8, 7 mm MgAc, 0.1 mm EGTA, 1 mm DTT, 0.25 mg·mL^−1^ BSA, 0.01 mg·mL^−1^ creatine kinase, 40 mm creatine phosphate, 4 mm ATP, 0.2 mm each of CTP, GTP and UTP, 100 μm of each dATP, dGTP and dTTP plus 50 μm dCTP in the presence of 0.1 μCi P32 α‐dCTP (3000 Ci·mmol^−1^, PerkinElmer, Waltham, MA, USA) and 250 ng ΦΧ174‐ssDNA template (0.76 nmol nucleotides, NEB) in 40 µL volumes. To compare the effect of Tag in the absence or presence of RPA, 0.5 µg of RPA was added to the assay as indicated. The assay mixtures were assembled on ice, and the reactions were started by adding 10 ng of human Pol‐prim. The reaction mixtures were incubated for 1 h at 37 °C and then spotted on glass fibre filters, which were submerged in ice‐cold 10% trichloroacetic acid for 10 min as previously described [41]. The filters were washed four times with 1 M HCl, twice with ethanol, and dried. Then, the incorporation of radioactive dNMPs was measured using a TriCarb scintillation counter (PerkinElmer). All experiments were performed in triplicates and the median and standard deviation are presented. Statistical comparisons were performed using paired Student’s *t*‐test. A *P* value of < 0.05 was considered to be statistically significant.

## Results and Discussion

### The dimerisation interface of the Tag helicase domain is distinct from the Pol α‐p68 subunit recognition site

**Fig. 1:**
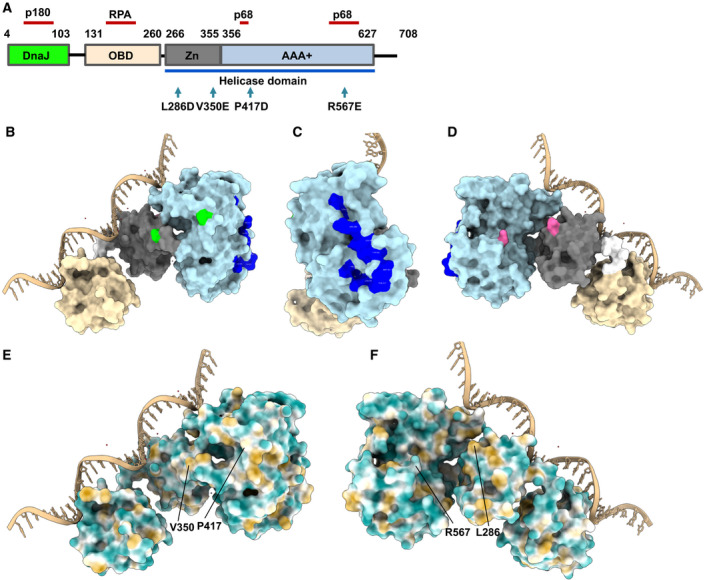
Structure of SV40 Tag. (A) SV40 Tag consists of four structural domains with different functions [6, 8]: the DnaJ domain (aa 4‐103, highlighted in green), the origin‐binding domain (OBD, aa 131‐260, shown in beige), Zn‐binding domain (aa 266‐355, presented in grey), and the AAA+ ATPase domain (aa 266‐355, light blue). The Zn and ATPase domain together form the helicase domain (aa 266‐627, highlighted by the blue line underneath the panel). In addition, the RPA binding site of Tag [22], the Pol‐prim p68 subunit binding site (p68) [47, 48], and the Pol α‐p180 interacting residues [27] are highlighted by red‐brown lines. The mutated Tag residues (V350, P417, L286 and R567) are shown in the linear Tag model by arrows. (B–D) Localisation of mutated residues and p68 binding sites in Tag_131‐627_. The Tag structure is derived from 4GDF (PDB) and the protein structure, chain A, is presented using the program chimerax [36]). Tag OBD is shown in beige, its Zn‐binding domain is grey, and its ATPase domain is light blue. The connecting residues between OBD and Zn domain, aa 261 to 265, are shown in bright white. The residues of the Pol‐prim p68 binding site are shown in dark blue. The modelled ssDNA is shown in light brown. (B) The residues V350 and P417 of Tag_131‐627_, which are mutated in the Tag variant M1, are shown in green. (C) The Tag_131‐627_ protein structure seen in panel B is rotated by 90° around the *y*‐axis and the p68 binding residues are seen in dark blue. (D) The protein‐ssDNA complex presented in panel B rotated by 180° shows the residues L286 and R567 of Tag_131‐627_, that are mutated in the Tag variant M2, highlighted in magenta. These three views of Tag_131‐627_ show that the M1 and M2 mutations are located on opposite sides of Tag_131‐627_ and that both do not overlap with the Pol‐prim p68 binding site of Tag_131‐627_. These four residues (V350, P417, L286 and R567) are required for SV40 Tag dimerisation and oligomerisation [29]. (E, F) Hydrophobic surface map of a SV40 Tag monomer, containing OBD and helicase domain (PDB: 4GDF: protein chain A, modelled in complex with ssDNA (chimerax). The MLP map of Tag_131‐627_ is presented with nonprotein atoms being ignored. The results are shown with colouring ranging from dark cyan (most hydrophilic) to white (MLP = 0) to dark goldenrod (most lipophilic). In panel E, the residues V350 and P417, which are changed to glutamate and aspartate, respectively, in the Tag_131‐627_ variant M1, are highlighted. In panel F, the Tag_131‐627_ molecule is rotated by 180° around the y‐axis, and the residues L286 and R567, which are mutated to aspartate and glutamate, respectively, in the Tag_131‐627_ variant M2, are indicated.

The multifunctional full‐length SV40 Tag has four structural domains with different roles in viral replication and cellular transformation (Fig. 1A, summarised in [18, 42, 43]). Tag is composed of the DnaJ domain at the N terminus, followed by the origin‐binding domain (OBD), the Zn‐binding and the ATPases associated with diverse cellular activities (AAA+) ATPase domain with the Zn‐binding and ATPase domain together forming the helicase domain (Fig. 1A). To study the function of monomeric in contrast to oligomeric Tag, mutations were introduced into the OBD‐helicase‐domain‐containing Tag_131‐627_. These mutations result in the monomeric Tag variants Tag_131‐627_(V350E/P417D) (M1) and Tag_131‐627_(L286D/R567E) (M2) [29, 30]). The M1 and M2 mutations are localised on opposite sides within the Tag helicase domain (Fig. 1B–D and Movie S1). The L286D and V350E mutations are localised in the Zn domain, whereas P417D and R567E are found in the ATPase domain (Fig. 1A–D and Movie S1). Each of these double mutations prevents the protein variants M1 and M2 to dimerise or oligomerise ([29, 30] and this study, see below) whereas mixing M1 and M2 results in M1‐M2 dimers as previously shown by size‐exclusion chromatography (SEC) [29]. Interestingly, the residues of Tag, which form the Pol α‐p68 subunit binding site, are not overlapping with these mutant residues (Fig. 1B–D and Movie S1), so that in principle, Pol α‐p68 interactions with Tag should not be affected in either of the two variants.

Analyses of the molecular lipophilicity potential (MLP) of Tag_131‐627_ (Fig. 1E–F) demonstrate that the regions surrounding L286, V350 and P417 are mainly lipophilic with adjacent hydrophilic residues. In contrast, R567 and the surrounding region are preferentially hydrophilic. Looking at the electrostatic potential (Fig. S1) of the areas surrounding these mutations, these range from slightly negative to neutral for L286, V350 and R567, and neutral to slightly positive for the area surrounding P417. Introducing aspartates and glutamates into these regions changes the environment of the mutant residues to more negative and hydrophilic, which may be responsible for the observed lack of oligomerisation of the Tag variants M1 and M2 [29, 30].

**Fig. 2:**
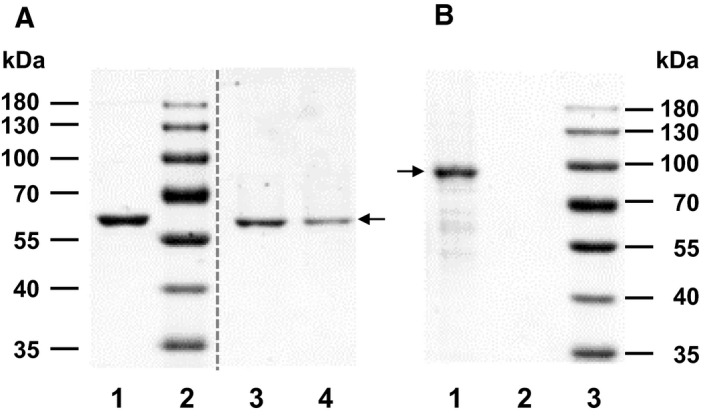
SDS Gels of SV40 Tag variants. (A) The SV40 Tag variants, Tag_131‐627_ wt, M1, and M2 were produced in *E. coli* and purified as described [30]. For SDS/PAGE analysis, 2.5 μg of wt Tag_131‐627_ (lane 1), 1.5 μg M1 (lane 3) and 1 μg of M2 (lane 4) were applied. The gel shows highly pure protein with a molecular weight of ~ 62 kDa (arrow). The vertical dashed line indicates the position where intervening lanes of the protein gel between the two spliced lanes were removed. (B) Affinity‐purified full‐length Tag_1‐708_ (1.5 μg) in lane 1 showed one main band (arrow) with a molecular weight of ~ 82 kDa in SDS/PAGE analysis, plus a few additional, smaller protein bands, which were mainly protein degradation products of Tag_1‐708_ since the majority of these bands were recognised by Tag‐specific polyclonal antiserum raised against full‐length Tag_1‐708_ (data not shown, [55]). Lane 2 is empty and lane 3 contains the molecular weight marker. The proteins presented in the gel images are representatives of at least two independent expressions and purifications.

To study the function of Tag in the initiation of Okazaki fragment synthesis, we expressed and purified different Tag variants: full‐length Tag, which forms monomers and oligomers in solution [30, 44, 45, 46], a shortened version wt Tag_131‐627_, and Tag_131‐627_ monomeric variants M1 and M2 (Fig. 2). The SDS gel of Tag variants wt Tag_131‐627_, M1 and M2, showed a single protein band at ~ 62 kDa in each lane (Fig. 2A, lanes 1, 3 and 4, respectively) demonstrating > 95% purity. Full‐length Tag (~ 82 kDa, Fig. 2B, lane 1) was also highly pure and from the SDS gel it was estimated that the protein used in the biochemical experiments was ~ 95% pure. In agreement with previous results [29], M1 and M2 proteins exclusively formed monomers in solution, whereas affinity‐purified wt Tag_131‐627_ was separated into hexameric and monomeric forms, as determined by SEC using a Superdex 200 16/600 column similar as described earlier [29] (data not shown).

### Tag variants wt Tag_131‐627_ and M2 bind to single‐stranded DNA

**Fig. 3:**
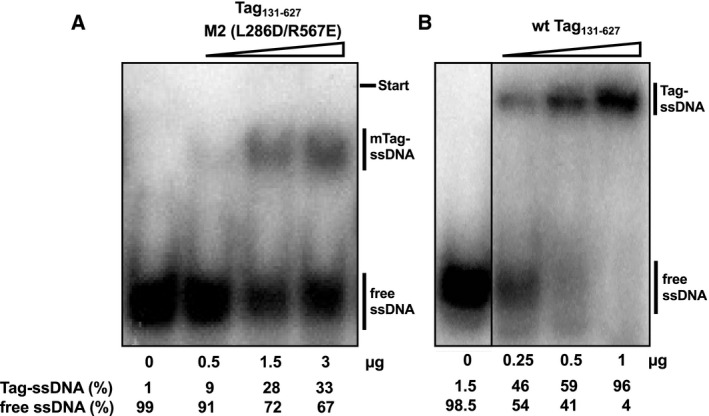
Monomeric SV40 Tag variant M2 binds to ssDNA. The SV40 Tag variants consist of the origin‐binding and helicase domains, aa 131‐627. Wild‐type (wt) Tag_131‐627_ and mutant M2 that abolishes Tag oligomerisation (L286D/R567E) [29, 30] were analysed for ssDNA binding using electrophoretic mobility shift assays (EMSAs). (A) Increasing amounts of the purified M2 protein as indicated were incubated with radioactively labelled ssDNA (GAPDH ssDNA primer shown in the first lane without protein added, see Materials and methods) and then loaded on a native polyacrylamide gel for EMSAs. The bars on the right indicate free ssDNA and monomeric Tag (mTag)‐ssDNA complexes (B) Increasing amounts of purified wt Tag_131‐627_ as indicated were incubated with radioactively labelled ssDNA (GAPDH ssDNA primer running on the same gel is shown in the first lane in the absence of proteins) and subjected to EMSAs. The bars on the right highlight the free ssDNA and Tag‐ssDNA complexes for wt Tag_131‐627_. The relative amounts of bound and free DNA after subtracting the relevant background intensities are shown underneath the autoradiography. The EMSAs were performed in three independent experiments and the gel shift images are representatives of these DNA binding experiments.

The initiation of Okazaki fragment synthesis requires the physical and functional interactions of at least three proteins (Pol‐prim, RPA and Tag) with each other and with ssDNA templates produced during viral DNA replication by hexameric Tag helicase. RPA has a high affinity to ssDNA and inhibits DNA primase; however, this inhibition is reversed by SV40 Tag with all necessary auxiliary functions located in the OBD and helicase domain, amino acids 131–627 [30]. The interactions of Tag with RPA, Pol‐prim and ssDNA are believed to be necessary to stimulate the initiation of DNA synthesis on an RPA‐bound ssDNA template [17, 19, 20, 22, 47, 48]. It is generally thought that full‐length Tag functions as a double hexamer in replication initiation [8, 17, 18, 19, 20, 22, 47, 48]. In contrast, the M1 and M2 variants do not oligomerise and form exclusively monomers as previously described [29]. To analyse the ssDNA binding activity of recombinant proteins, wt Tag_131‐627_ and mutant protein M2 were incubated with ssDNA, and free DNA and protein‐ssDNA complexes were separated using native PAGE EMSAs (Fig. 3). In these EMSA studies, M2 and wt Tag_131‐627_ showed concentration‐dependent binding to the ssDNA substrate (Fig. 3A,B, respectively). Interestingly, the M2 variant bound ssDNA with lower affinity than that of the parent protein wt Tag_131‐627_. In experiments performed in parallel, the latter shifted the DNA to ~ 50% with 0.5 µg and nearly fully shifted the ssDNA with 1 μg of protein, whereas 3 µg of M2 protein only bound 33% of the ssDNA (Fig. 3A,B).

### Mixing monomeric Tag variants M1 and M2 yields dimers capable of binding ssDNA

Previously, it was shown that full‐length Tag_1‐708_ binds to ssDNA in an equilibrium of monomeric and oligomeric forms whereas the shortened Tag variant wt Tag_131‐627_ (consisting of the origin‐binding and the helicase domain) forms predominantly oligomeric complexes with ssDNA ([30] and Fig. 3B). Monomeric M2 binds to ssDNA as a monomer as shown by EMSA (Fig. 3A). Since purified M1 and M2 proteins form monomers and, after mixing, dimers in solution as well as on dsDNA sequences containing an SV40 origin of replication [29], we further tested whether pre‐mixed M2 and M1 interact also with ssDNA as a dimer. To analyse the oligomeric state of proteins bound to DNA, AFM imaging is an excellent method [30, 49, 50]. In contrast to EMSAs, which also allow differentiation between monomeric and oligomeric Tag‐DNA complexes [30], AFM directly determines the size of these complexes on a single‐molecule level to more unambiguously distinguish between monomeric, dimeric and oligomeric protein forms. AFM imaging was therefore selected to characterise and compare the protein‐ssDNA complexes that are formed by full‐length Tag, wt Tag_131‐627_, monomeric M2 protein and M1‐M2 proteins.

**Fig. 4:**
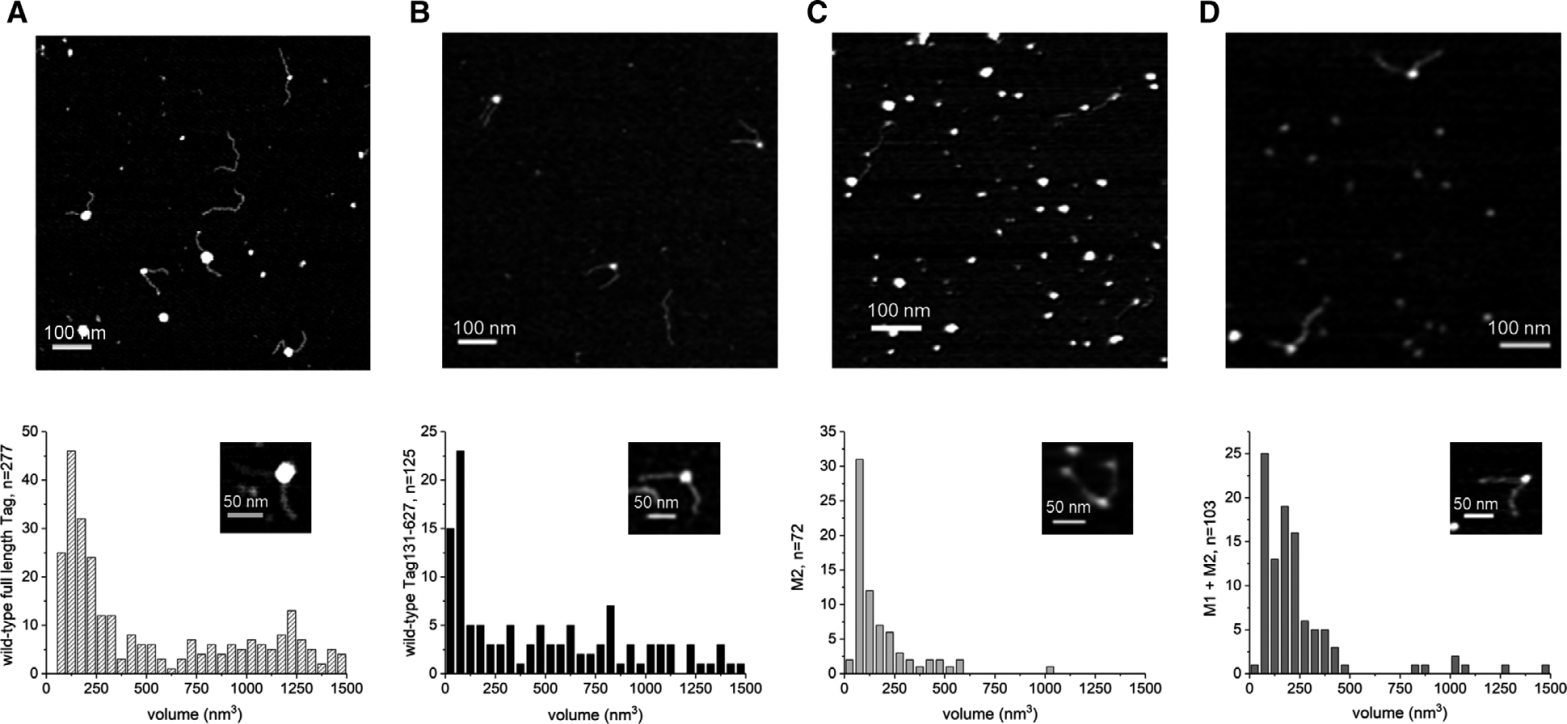
The SV40 Tag proteins M1 and M2 form dimers and bind to ssDNA as dimers. AFM imaging was used to study the protein complexes bound to ssDNA (48 nt at the centre of ~ 500 bp fragments) in the presence of full‐length Tag and different Tag_131‐627_ variants. Incubations were carried out at 500 nm protein concentration and samples were subsequently crosslinked with 0.1% glutaraldehyde and diluted for deposition onto mica substrate for AFM imaging. The panels show ssDNA in the presence of (A) full‐length Tag (Tag_1‐708_), (B) wt Tag_131‐627_, (C) M2, and (D) M1 plus M2. The bottom panels show volume distributions of peaks on the central ssDNA in the substrates, with an inset showing an enlarged representation of a representative substrate molecule for each sample (scale bars 50 nm). The top panels show representative AFM images for the different samples, with scale bars representing 100 nm. The negative control of DNA fragments containing ssDNA at 50% DNA length in the absence of protein is presented as Fig. S2. Gaussian fits to the low volume regime (< 500 nm^3^) are shown for A–D in Fig. S3. The DNA binding experiment were performed in at least two independent experiments and the AFM images and quantifications are representatives of these experiments.

For these experiments, 500 nm of protein (full‐length Tag, wt Tag_131‐627_ and M2 protein), or a mixture of 250 nm of each M1 and M2 were incubated with ssDNA. AFM imaging in the absence of protein showed small peaks (~ 50 nm^3^, see Fig. S2 right panel) for ssDNA stretches of 48 nt at the centre of long (~ 500 bp) DNA fragments that likely result from ssDNA superstructures. In addition to these ssDNA peaks, AFM volume analyses on samples of ssDNA and wt Tag_131‐627_ revealed large complexes with volumes ≥ 500 nm^3^ on the ssDNA stretches (Fig. 4B) that correspond to oligomeric states of Tag_131‐627_ (hexamers, double hexamers and some even larger complexes). These large volume states for wt Tag_131‐627_ were similar as for full‐length Tag (Fig. 4A, [30]). Interestingly, Gaussian fits to the small volume regime (< 500 nm^3^, Fig. S3A,B) indicated monomers (~ 100 nm^3^) and dimers (~ 200 nm^3^) for the full‐length protein, while for wt Tag_131‐627_ these were mostly absent. The small volume peak at ~ 50 nm^3^ for wt Tag_131‐627_ is consistent with and likely reflects the background of unbound ssDNA (compare to Fig. S2). In contrast to wt Tag_131‐627_, AFM volume analyses for the monomeric M2 mutant showed protein complexes on ssDNA with almost exclusively volumes of ~ 90 nm^3^ (Figs 4C and S3C) suggesting that M2 binds to the DNA as a monomer. Mixing the monomeric variants M1 and M2 (250 nm each) together in the presence of ssDNA containing DNA substrate resulted in complex volumes at the central ssDNA stretches that were slightly larger than those of M2 alone with ssDNA (Fig. 4D). Gaussian fits to the data in the small volume regime revealed two states with ~ 90 nm^3^ and ~ 180 nm^3^, consistent with monomers and dimers (Fig. S3D). One has to take into account that the dimer peak volumes contain two molecules of Tag (one M1 plus one M2 molecule), whereas the monomer peak has one molecule of either M1 or M2, which cannot be discriminated (See Fig. S3E,F for volumes of the individual proteins M1 and M2 in the absence of DNA). The monomeric population very likely consists of ~ 50% M1 and ~ 50% M2. It is important to note that the volume distribution for M1–M2 complexes on ssDNA contains barely any high molecular weight complexes with volumes ≥ 500 nm^3^ consistent with the lack of the second interaction interface in both monomers leading to abolished oligomerisation. Taken together, the EMSA and AFM data suggest that both M1 and M2 bind to ssDNA as monomers and, if mixed together, they form dimers on ssDNA. The latter is consistent with previous data that M1–M2 form dimers that bind to dsDNA containing an origin of SV40 and can be crystallised [29] suggesting that the purified recombinant proteins are active in biochemical assays.

### The Tag monomer M2 but not the M1 monomer stimulates primase‐initiated DNA synthesis on ssDNA

**Fig. 5:**
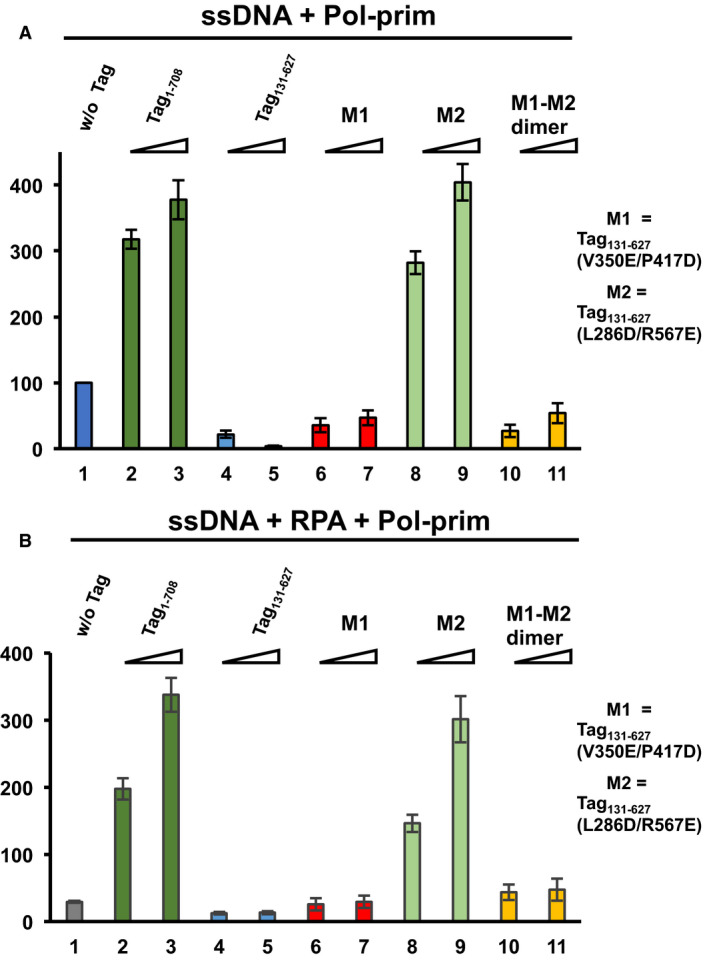
The SV40 Tag helicase domain regulates Tag oligomerisation and functional interactions during Okazaki fragment synthesis. DNA synthesis on ssDNA by Pol‐prim was measured in the absence (A) and presence of RPA (B) to determine the stimulatory activity of SV40 Tag‐derived proteins in these biochemical test systems. Pol‐prim‐dependent DNA synthesis on ssDNA is shown for increasing amounts of Tag‐derived proteins (0.3 and 0.6 µg). (A) DNA synthesis on ssDNA by 0.1 unit of Pol‐prim alone (without (w/o) Tag) was measured and set to 100% (panel A, blue column 1). Columns 2 and 3 (dark green) present the stimulation of Pol‐prim by 0.3 and 0.6 µg of full‐length Tag (Tag_1‐708_), respectively. Columns 4 and 5 (light blue) show Pol‐prim activity in the presence of the Tag variant wt Tag_131‐627_ (0.3 and 0.6 µg, respectively) consisting of the OBD and the helicase domain. Furthermore, DNA synthesis on ssDNA by Pol‐prim in the presence of increasing amounts of Tag variant M1 (columns 6 and 7, 0.3 and 0.6 µg, respectively, red colour), M2 protein (columns 8 and 9, 0.3 and 0.6 µg, light green), and M1‐M2 dimer (columns 10 and 11, 0.3 and 0.6 µg, light orange) is presented. Full‐length Tag (consisting of monomers and oligomers [30, 46]) and the monomer mutant M2 stimulated Pol‐prim activity, whereas wt Tag_131‐627_, the monomeric mutant M1 and the M1‐M2 dimer inhibited Pol‐prim. (B) Adding 0.5 µg of RPA significantly reduced dNMP incorporation on ssDNA by Pol‐prim to 29% compared to Pol‐prim alone (compare panel A, column1 with panel B, grey column 1, indicated as w/o Tag above the column). Similar as in panel A, DNA synthesis by Pol‐prim on RPA‐bound ssDNA (0.5 µg of RPA) was determined in the presence of increasing amounts of the indicated Tag variants (0.3 and 0.6 µg). Pol‐prim DNA synthesis in the presence of full‐length Tag (columns 2 and 3, dark green), wt Tag_131‐627_ (columns 4 and 5, light blue), mutant M1 (columns 6 and 7, red colour), mutant M2 (columns 8 and 9, light green) and M1‐M2 dimer (columns 10 and 11, light orange) is shown. Full‐length Tag and monomeric Tag variant M2 stimulated the DNA synthesis, whereas wt Tag_131‐627_ inhibited DNA synthesis by Pol‐prim further in comparison to RPA alone (column 1). Monomeric Tag M1 neither stimulated nor inhibited the reaction under the condition tested, and the dimer M1+M2 slightly stimulated DNA synthesis on RPA‐ssDNA by Pol‐prim from 29% to ~ 45% (compare column 1 in panel B with columns 10 and 11). All experiments were performed in three independent experiments, and the average and standard deviation of dNMP incorporation relative to the incorporation of DNA synthesis by Pol‐prim alone are presented.

As shown above, Tag variants M1 and M2 as well as the M1–M2 dimer bind to ssDNA. Tag is a multifunctional protein, the molecular biological equivalent of a ‘Swiss Army knife’ [43]. The protein functions as transcription and replication factor. Its activities include origin‐binding, ATPase, helicase activity and regulatory functions, for example stimulation of DNA synthesis by Pol‐prim [17, 18, 19, 20, 40]. To investigate the role that Tag oligomerisation plays in the regulation of these functions, we analysed whether the monomeric Tag variants, M1 and M2, and the M1‐M2 dimer are able to stimulate primase‐dependent DNA synthesis by Pol‐prim on ssDNA (Fig. 5A). Consistent with previously published work [17, 19, 20, 30, 34, 40], full‐length Tag_1‐708_ stimulated the primase‐DNA polymerase reactions 3.2 to 4‐fold in this assay (compare columns 2 and 3 with column 1 in Fig. 5A). In contrast, adding wt Tag_131‐627_ (lacking the DnaJ chaperone domain and the host specificity region) to Pol‐prim resulted in inhibition of primase‐dependent DNA synthesis by Pol α in a concentration‐dependent manner by 78% and 96% compared to the DNA synthesis by Pol‐prim in the absence of Tag (Fig. 5A, compare columns 4 and 5 with column 1), similar to previously published data [30]. Interestingly, addition of Tag M1 to the assay reduced the incorporation of dNMPs by ~ 65% and 53% (Fig. 5A, compare columns 6 and 7 with column 1). Inhibition by the M1 monomer was, however, weaker than that by wt Tag_131‐627_, and it did not increase with increasing amounts of protein as seen for wt Tag_131‐627_. In complete contrast to M1, the Tag M2 monomer stimulated dNMP incorporation by Pol‐prim in the assay in a concentration‐dependent manner by factors of 2.8 and 4 for the same concentrations (0.3 μg and 0.6 μg, respectively) as used in the assays with M1 (Fig. 5A, compare columns 8 and 9 with column 1). These stimulations are similar as with full‐length Tag protein (compare column 8 with 2 and 9 with 3). Importantly, mixing the proteins M1 and M2, which together form dimers in solution and on ssDNA (see Fig. 4 and Ref. [29]), again resulted in an inhibition of the primase‐dependent DNA synthesis by 50–70% (Fig. 5A, compare columns 10 and 11 with column 1). This degree of inhibition is similar as that seen for M1 (compare columns 10 and 11 with columns 6 and 7) and again the inhibition is slightly weaker with higher protein concentrations, as for M1. These findings suggest that the mutation of Tag residues V350E and P417D in M1 have a dominant effect in the M1‐M2 dimer. Here, it is important to note that the M2 monomer of the dimer contains the wt residues V350 and P417 but they are involved in the dimerisation with M1 and thus masked in the complex. Therefore, these residues, V350 and P417, are not only important for the oligomerisation of Tag to assemble the hexameric helicase and the double‐hexamer at the SV40 origin of replication, but also for the physical and functional interactions of Tag with Pol‐prim to stimulate primase‐dependent DNA synthesis on ssDNA.

These findings prompted us to investigate whether similar results were found for the primase‐dependent DNA synthesis on RPA‐bound ssDNA, a general biochemical model system for the investigation of Okazaki fragment synthesis. As previously described [17, 19, 22, 30, 34, 40], RPA inhibits primase‐dependent DNA synthesis. In the assay, 0.5 µg of RPA reduced the DNA synthesis on ssDNA by ~ 70% in comparison to that on free ssDNA (Fig. 4, compare panel B column 1 to A column 1) comparable to previous results. Addition of full‐length Tag reversed the RPA‐dependent inhibition of primase‐ and DNA polymerase‐dependent DNA synthesis in a concentration‐dependent manner and stimulated the incorporation of dNMPs by factors of 7 and 11.5‐fold (for increasing concentrations) compared to reactions in the presence of RPA but absence of Tag (Fig. 5B, compare columns 2 and 3 to column 1). These findings are again in agreement with previously reported data [17, 19, 22, 30, 34, 40]. Similar to the full‐length Tag protein, M2 stimulated the dNMP incorporation in this assay by factors of 5 and 10.4 (for the same increasing concentrations) compared to in the presence of RPA but in the absence of Tag (compare columns 8 and 9 with column 1 in Fig. 5B). In contrast, both wt Tag_131‐627_ and the M1 variant did not stimulate the incorporation of dNMPs by Pol‐prim (compared to in the presence of RPA only, Fig. 5B, compare columns 4–7 with column 1). In fact, wt Tag_131‐627_ even inhibited DNA synthesis significantly [*P* < 0.01 for the lower wt Tag_131‐627_ concentration (comparison of column 4 to 1) and *P* < 0.025 for the higher wt Tag_131‐627_ concentration (comparison of column 5 to 1)]. Again, these results for wt Tag_131‐627_ are consistent with previous findings [30]. In contrast, M1 protein neither stimulated nor inhibited Pol‐prim‐dependent DNA synthesis on RPA‐ssDNA. Finally, addition of the dimer M1‐M2 resulted in a low but significant increase of dNMP incorporation by factors of 1.5–1.6 in the assay compared to RPA‐ssDNA (Fig. 5B, compare column 10 and 11 with column 1; comparison of column 10 to 1: *P* < 0.01, and 11 to 1: *P* < 0.025). Interestingly, the incorporation of dNMPs in the presence of M1‐M2 dimers and RPA, and that with M1‐M2 dimers but without RPA show only slight differences, which were not significant (*P* values > 0.05). These findings suggest that the dimer may reverse the inhibition by RPA but cannot stimulate Pol‐prim‐dependent DNA synthesis on ssDNA. In fact, the M1‐M2 dimer even inhibited dNMP incorporation on ssDNA templates in the absence of RPA (Fig. 5A). The slight increase of DNA synthesis on RPA‐bound ssDNA could thus be due to the dimer removing RPA from ssDNA without additional stimulation of Pol‐prim activity. Alternatively, the dimer may not be fully stable during the assay (see also monomer–dimer equilibrium in AFM analyses, Figs 3D and S3D) so that some free M2 protein might be available to interact with Pol‐prim and stimulate its RNA and DNA synthesis activities. It is expected that the double mutations of M1 and M2 have similar effects in full‐length Tag as in the Tag_131‐627_ background, that is interfering with the oligomerisation of Tag and interactions with other proteins. This hypothesis is supported by findings that M2 protein stimulates Pol‐prim on ssDNA and RPA‐bound ssDNA to a similar degree as full‐length Tag (Fig. 5A,B, compare columns 8 and 9 with columns 2 and 3).

**Fig. 6:**
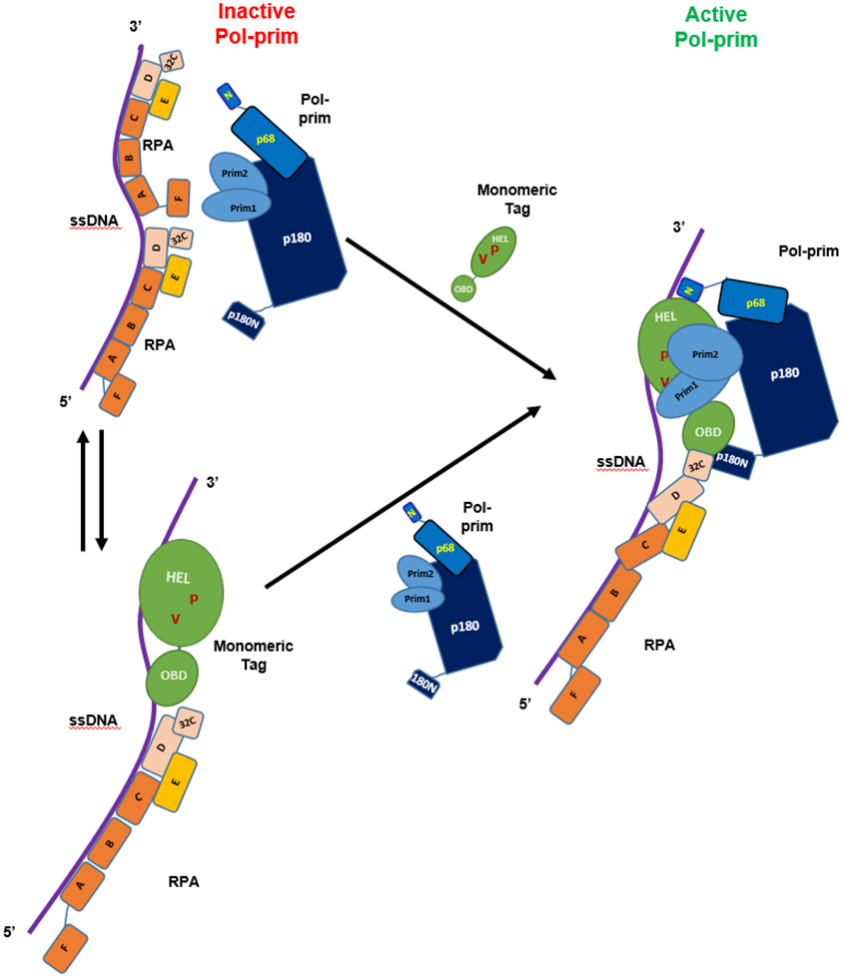
Model of SV40 Tag helicase domain regulating Tag oligomerisation and functional interactions during Okazaki fragment synthesis. RPA‐bound ssDNA is not a substrate for Pol‐prim‐dependent DNA synthesis and the primase does not start primer synthesis on RPA‐bound ssDNA. The addition of the auxiliary factor Tag is required that Pol‐prim is able to synthesise RNA and DNA on ssDNA in the presence of RPA. The components required for DNA synthesis are shown: ssDNA (single purple line); RPA (in brown the large subunit RPA70 with the OB‐fold domains A, B, C, and F; in beige middle subunit RPA32 containing N‐terminal phosphorylation sites, the central OB‐fold D and the protein interaction region RPA32C, and the small RPA14 subunit in orange consisting of the OB‐fold domain E [12, 13]); Pol‐Prim (four subunits: DNA polymerase [10, 56] subunit p180 in dark blue consisting of N‐terminal Tag interaction region (p180N) and the catalytic DNA polymerase and additional protein interaction sites, with its core regulatory subunit p68 in blue, consisting of the p68 N‐terminal region (p68N) that interacts with SV40 Tag and the remaining p68 residues necessary for DNA replication [48]; and in light blue the two primase subunits p48 (Prim 1) containing the catalytic site and the large primase subunit p58 (Prim2) that interacts with the large subunit p180); and the auxiliary factor Tag (green, showing only the minimal regions required for the stimulation of Pol‐prim, the OBD and the helicase domain with aa V350 and P417 necessary for the stimulation shown as V and P and highlighted in red‐brown). Interactions between Pol‐prim and Tag are indicated: p68N (N) with the AAA+ domain of Tag (Fig. 1B–D), p180N with the OBD (Fig. S4A). Furthermore, our data suggest that interactions of Pol‐prim with Tag residues V350 and/or P417 are necessary for Pol‐prim stimulation.

These findings suggest that multiple physical interactions between Tag and Pol‐prim are required to allow the initiation and elongation of DNA synthesis on RPA‐bound ssDNA (see model in Fig. 6). Importantly, RPA and Pol‐prim physically interact with the OBD and the helicase domain of Tag (Figs 1A–D and S4, and summarised in the model, Fig. 6; [22, 47, 48]). These findings are reminiscent of the replacement of RPA from resected ssDNA ends during homologous recombination where Rad52 interacts with RPA32C and destabilises the RPA‐ssDNA complexes so that Rad51 can bind to ssDNA establishing Rad51‐ssDNA filaments needed for strand invasion [24]. Here, we propose that stimulation of Pol‐prim requires the interface containing the residues V350 and P417 that is accessible in Tag monomers, but not in (closed) hexamers [29, 51]. In our model, enhanced activity by full‐length Tag is due to the monomers seen in addition to oligomers for the full‐length protein in AFM volume analyses on ssDNA (Figs 4A and S3A). In contrast, wt Tag_131‐627_ showed predominantly oligomers on ssDNA (Figs 4B and S3B, [30]), in which this interface containing the V350 and P417 residues is covered [51], consistent with the lack of Pol‐prim stimulation by this Tag variant. Docking of p68 at its binding site, which is localised in the ATPase domain of Tag is fully separated from Tag oligomerisation residues (Fig. 1B–D; [48]). These p68‐Tag interactions are necessary for the Okazaki fragment synthesis and may serve as an anchoring point to allow the rest of the Pol‐prim complex to contact binding sites located within this V350 and P417 containing interface, and these latter interactions may be required for loading of Pol‐prim onto the ssDNA by Tag. The details of the involved interactions are summarised in our model (Fig. 6). It is interesting to note that the high affinity binding of Tag to ssDNA seems not to be required for the stimulation since the monomer M2 has a lower affinity to ssDNA than its parent protein Tag_131‐627_, which inhibits Pol‐prim‐dependent DNA synthesis on ssDNA and RPA‐bound ssDNA templates as discussed above (Fig. 5, panels A and B). However, multiple protein–protein interactions of Tag with Pol‐prim and RPA may be required. In addition to the previously proposed binding site of the N‐terminal peptide of p180 (p180N, aa195‐313 [52]) in the Tag DnaJ domain a second p180 binding site in Tag has been suggested to localise in the central part of Tag (Fig. S4A,B, [22, 27, 28]). Molecular docking of p180N and SV40 Tag shows that p180N may bind to Tag OBD, which in turn suggests that the primase subunits may interact with the protein surfaces surrounding V350 and/or P417 on the neighbouring helicase domain (summarised in the model Fig. 6) and these interactions support the primase activity during RNA synthesis on ssDNA. Interestingly, the predicted p180N binding region in Tag OBD overlaps with the interaction interface of Tag OBD and RPA32C (Fig. S4A,C, summarised in Fig. S4, panel D). However, molecular docking suggests multiple possible arrangements for p180N on Tag OBD (Fig. S4A) so that simultaneous binding of Tag OBD to p180N and RPA32C could be possible (see model Fig. 6). The binding of p180N to Tag OBD may even be necessary to stabilise the association of Tag OBD to RPA32C since the affinity of Tag OBD to bind to RPA32C is relatively weak [22]. These multiple interactions within the initiation complex for Okazaki fragment synthesis consisting of Pol‐prim, Tag and RPA would then in turn allow primase to initiate RNA synthesis and elongate the first dinucleotide. Alternatively, binding of p180N to Tag OBD may serve to interrupt the RPA32C‐Tag interaction releasing the Tag‐Pol‐prim complex for DNA translocation and primer synthesis activity.

In summary as shown in Fig. 6, multiple, highly coordinated protein–protein and protein‐DNA interactions of Tag direct Pol‐prim either on RPA‐bound ssDNA or on RPA‐Tag‐bound ssDNA to the template to initiate DNA synthesis. It is important to note that using the monomeric form of Tag for viral DNA replication functions opens the opportunity for Tag to have more residues available to interact with other replication factors (Figs 6 and S4). Additionally, shifting the equilibrium between monomeric Tag and hexameric Tag forms would allow to modulate these interactions for regulation of Tag‐specific protein interactions depending on the oligomerisation state of the protein, which is at least in part regulated by phosphorylation [53, 54].

## Conflict of interest

The authors declare no conflict of interest.

## Author contributions

HPN and IT conceived and designed the project, NOO, FS, IT and HPN acquired the data, NOO, IT and HPN analysed and interpreted the data and wrote the paper.

## Supporting information


**Fig. S1.** Electrostatic surface map of the SV40 T antigen OBD and helicase domain associated with ssDNA.
**Fig. S2.** Negative control of DNA fragments containing ssDNA at 50% DNA length in the absence of protein.
**Fig. S3.** Gaussian fits to AFM volumes in the small volume regime (< 500 nm^3^).
**Fig. S4.** Docking of p180N and SV40 Tag.

**Movie S1.** Localisation of mutated residues and p68 interacting binding sites in Tag_131‐627_.

## Data Availability

The supporting data are contained in the manuscript as Supplementary Information including a Movie S1.
