# Supplementary material for: SV40 T antigen helicase domain regions responsible for oligomerisation regulate Okazaki fragment synthesis initiation
Source: FEBS Open Bio. 2022 Feb 7;12(3):649–63. doi: 10.1002/2211-5463.13373 (PMC8886539; doi:10.1002/2211-5463.13373)
Supplement: Supplementary file 1 — Fig. S1. Electrostatic surface map of the SV40 T antigen OBD and helicase domain associated with ssDNA. Fig. S2. Negative control of DNA fragments containing ssDNA at 50% DNA length in the absence of protein. Fig. S3. Gaussian fits to AFM volumes in the small volume regime (< 500 nm3). Fig. S4. Docking of p180N and SV40 Tag. [file FEB4-12-649-s001.pdf]

Simian virus 40 T antigen helicase domain regions responsible for oligomerisation regulate Okazaki fragment synthesis initiation

Onwubiko NO[1], Scheffel F[2], Tessmer I[2*], Nasheuer HP[1*]

[1]Biochemistry, School of Biological and Chemical Sciences,
Biomedical Sciences Building, NUI Galway, New Castle Road, Galway, H91 W2TY, Ireland
[2] Rudolf Virchow Center for Experimental Biomedicine, University of Würzburg, Josef Schneider Strasse 2, D-97080 Würzburg, Germany

*Correspondence should be addressed to:
Heinz Peter Nasheuer (heinz.nasheuer@nuigalway.ie) or
Ingrid Tessmer (Ingrid.tessmer@virchow.uniwuerzburg.de)

Supplemental Data

The interactions of SV40 Tag with ssDNA, RPA, and Pol-prim require specific residues of the protein which have been model using the ChimeraX program and the hydrostatic surface map plus a movie localising all four mutants and the Pol-prim p68 binding site are presented (Figure S1 and Movie M1, [1]). To understand the interactions of Tag and Tag variants with ssDNA, Atomic Force Microscopy image analyses were performed which provide deeper insights into these Tag-ssDNA complexes (Figures S2 and S3, [2]). Moreover, using previously published data and structural data docking models were established for the binding to the N-terminal peptide of the p180 subunit of Pol-prim on Tag. Additionally, the NMR structure of the Tag OBD with RPA32C is shown for comparison (Figure S4, A to C [3-5],). A revised model for the localisation of cellular replication factor binding sites on Tag is also presented (Figure S4, D [6-10]).

N. Onwubiko; Supplemental Figure S1

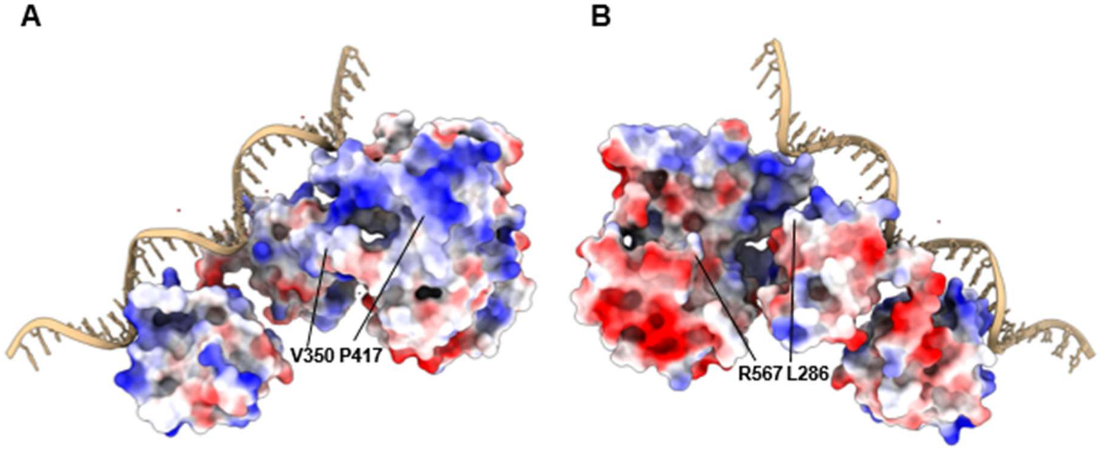

Supplemental Figure S1 Electrostatic surface map of the SV40 T antigen OBD and helicase domain associated with ssDNA. Panels A and B show the electrostatic surface map of a single SV40 T antigen monomer (OBD and helicase domain, PDB: 4GDF, protein chain A, modelled with ssDNA in ChimeraX). The electrostatic surface maps of the two sides of $Tag_{131-627}$ are presented with non-protein atoms being ignored. Electrostatic potential ranges from red for negative to white (neutral) to blue for positive electrostatic potential. In panel A the residues V350 and P417, which are changed to glutamate and aspartate, respectively, in the $Tag_{131-627}$ variant M1, are indicated. In panel B, the $Tag_{131-627}$ molecule is rotated by 180° around the y axis, and the residues L286 and R567, which are mutated to aspartate and glutamate, respectively, in the $Tag_{131-627}$ variant M2, are indicated.

N. Onwubiko; Supplemental Figure S2

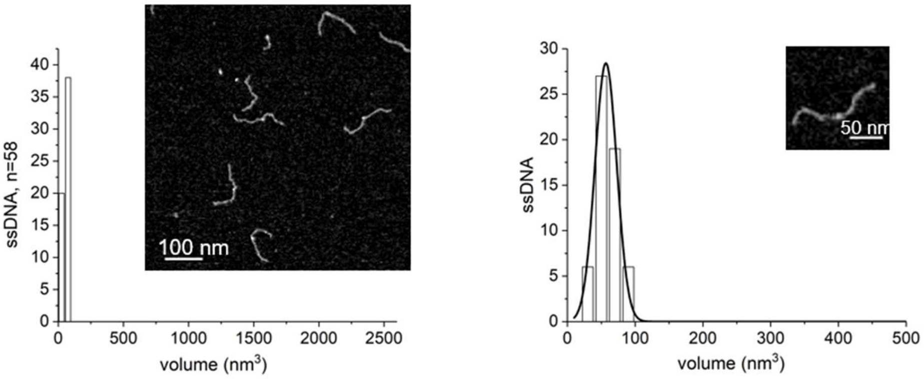

Supplemental Figure S2: Negative control of DNA fragments containing ssDNA at 50% DNA length in the absence of protein. Left: volume distribution of peaks at ssDNA position (inset: example image, scale bar 100 nm). Right: volume distribution in the small volume regime (< 500 nm^3, inset: example image, scale bar 50 nm). A Gaussian fit to the distribution shows a maximum at 57 nm^3 for the ssDNA superstructure peaks in the absence of protein

N. Onwubiko; Supplemental Figure S3

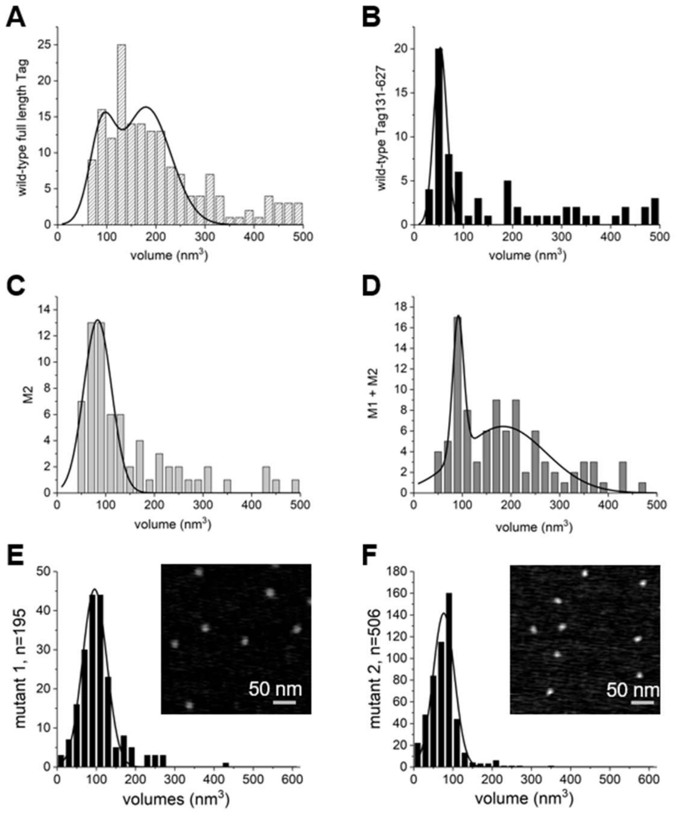

Supplemental Figure S3: Gaussian fits to AFM volumes in the small volume regime (< 500 nm^3). (A) Full length Tag complexes on ssDNA show maxima at 90 nm^3 and 180 nm^3, as determined from Gaussian fits to the data. **(B)** $Tag_{131-627}$ complexes show an absence of monomeric or dimeric volumes in the small volume regime. The Gaussian fit indicates a maximum at a volume of 53 nm^3, consistent with ssDNA super-structures in the absence of protein (see Supplemental Figure S2). **(C)** M2-ssDNA complexes have a maximum at 83 nm^3 consistent with monomeric Tag. **(D)** M1+M2-ssDNA complexes have Gaussian fit maxima at 91 nm^3 and 184 nm^3 consistent with monomeric and dimeric Tag, respectively. **(E.F)** M1 (E) and M2 (F) volumes in the absence of DNA with maxima at ~90 nm^3. The insets show exemplary images (scale bars 50 nm).

N. Onwubiko; Supplemental Figure S4

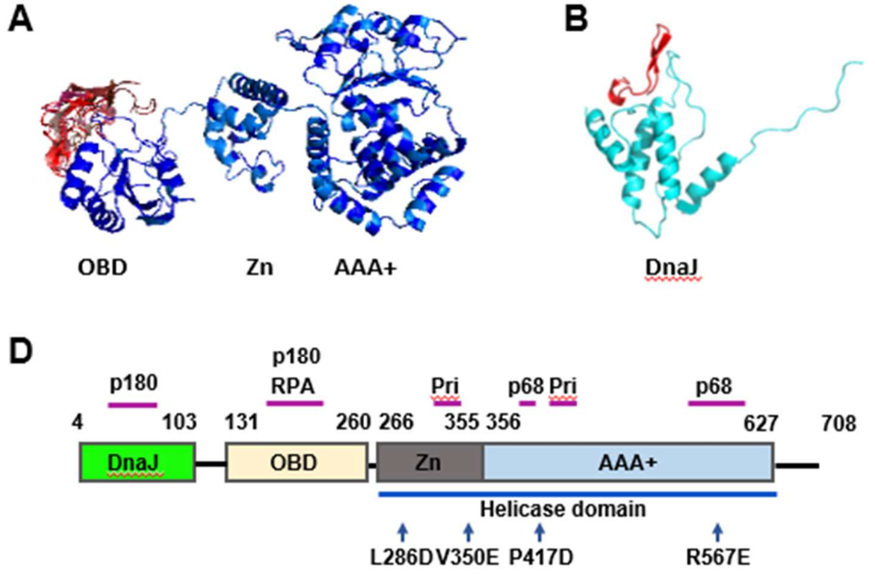

Supplemental Figure S4: Docking of p180N and SV40 Tag. (A) The interaction interface of Tag (helicase and OBD domains, in blue, PDB 4GDF) and the peptide of the N-terminal sequence of p180 in Pol-prim (p180N, aa195-313 [7], in red) as suggested by docking. The five energetically most favourable orientations for the p180N peptide are shown in different shades of red, and all reveal interactions with the same residues in Tag: R154, R202, R204. The interaction interfaces show an area of 1100-1500 Å^2 depending on the orientation of p180N peptide. **(B)** Model of the interaction interface (~1,400 Å^2) of the DnaJ domain of Tag (cyan, PDB 1GH6) and the p180N peptide (red). Modelling was performed using the Haddock web server [3,4] with active and passive residues on Tag helicase and OBD domains or Tag DnaJ domain assigned by Whiscy [5]. The p180N peptide structure used for docking has been modelled using SwissModel (swissmodel.expasy.org). **(C)** NMR structure of the complex of RPA32C (green) with the Tag OBD domain (PDB 1Z1D). Interactions from residues in Tag (R154, R202, and R204 [6]) display overlap with the predicted binding site in the Tag OBD for p180N shown in (A). The binding interface area was reported to be ~1,150 Å^2 and the K_D of the interaction to be moderate with ~60 μM [6]. **(D)** Model of Tag with binding sites for Pol-prim subunits and RPA32 (purple lines) including published sites shown in Figure 1A and those predicted by the current studies. The locations of the amino acid mutations in M1 and M2 are indicated by arrows.

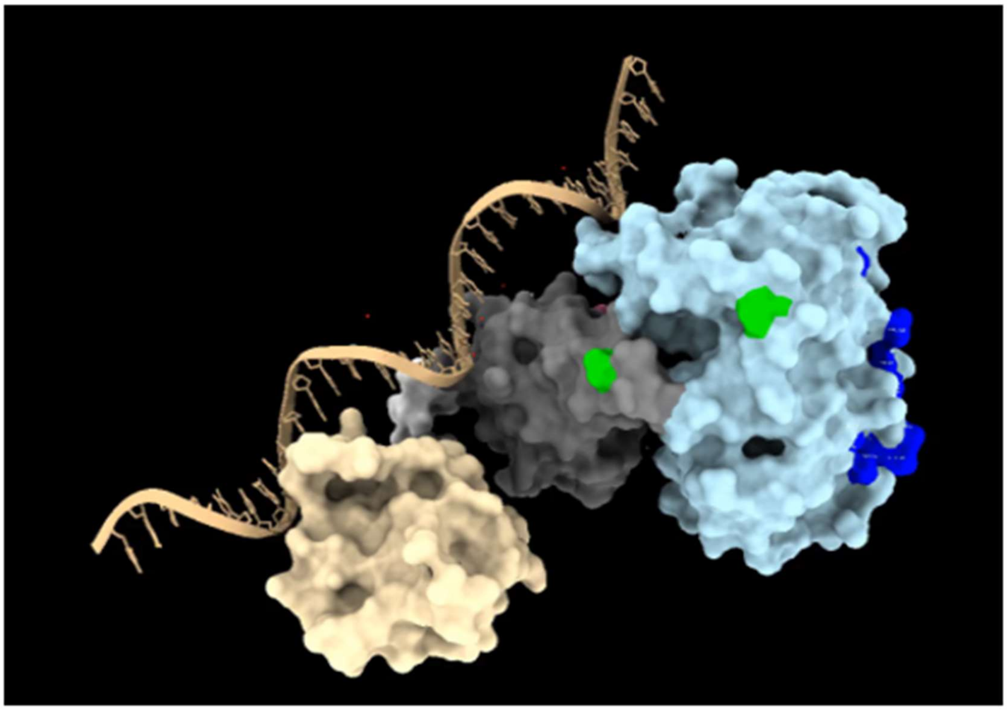

Supplemental Movie SM1: Localisation of mutated residues and p68 interacting binding sites in Tag131-627. The Tag structure is derived from 4GDF (PDB). Tag OBD is shown in beige, its Zn-binding domain is grey, and its ATPase domain is light blue. The connecting residues between OBD and Zn domain, aa 261 to 265, are highlighted in white whereas the residues of the Pol-prim p68 binding site are in dark blue. The modelled ssDNA is shown in light brown. The residues V350 and P417 of $Tag_{131-627}$, which are mutated in the Tag variant M1, are shown in green whereas the residues L286 and R567 of $Tag_{131-627}$ are shown in dark turquoise. These views of $Tag_{131-627}$ show that the M1 and M2 mutations locate on opposite sites of $Tag_{131-627}$, and that both do not overlap with the Pol-prim p68 binding site of $Tag_{131-627}$ described by Zhou et al. [10].

References:

[1] Pettersen, E., Goddard, T., Huang, C., Meng, E., Couch, G., Croll, T., Morris, J. and Ferrin, T. (2021). UCSF ChimeraX: Structure visualization for researchers, educators, and developers. . Protein Sci 30, 70-82.

[2] Onwubiko, N.O., Borst, A., Diaz, S.A., Passkowski, K., Scheffel, F., Tessmer, I. and Nasheuer, H.P. (2020). SV40 T antigen interactions with ssDNA and replication protein A: a regulatory role of T antigen monomers in lagging strand DNA replication. Nucleic Acids Res 48, 3657-3677.

[3] Dominguez, C., Boelens, R. and Bonvin, A.M.J.J. (2003). HADDOCK: A Protein−Protein Docking Approach Based on Biochemical or Biophysical Information. Journal of the American Chemical Society 125, 1731-1737.

[4] van Zundert, G.C.P. et al. (2016). The HADDOCK2.2 Web Server: User-Friendly Integrative Modeling of Biomolecular Complexes. Journal of Molecular Biology 428, 720-725.

[5] de Vries, S.J., van Dijk, A.D.J. and Bonvin, A.M.J.J. (2006). WHISCY: What information does surface conservation yield? Application to data-driven docking. Proteins: Structure, Function, and Bioinformatics 63, 479-489.

[6] Arunkumar, A.I., Klimovich, V., Jiang, X., Ott, R.D., Mizoue, L., Fanning, E. and Chazin, W.J. (2005). Insights into hRPA32 C-terminal domain--mediated assembly of the simian virus 40 replisome. Nat Struct Mol Biol 12, 332-9.

[7] Dornreiter, I., Copeland, W.C. and Wang, T.S. (1993). Initiation of simian virus 40 DNA replication requires the interaction of a specific domain of human DNA polymerase alpha with large T antigen. Mol Cell Biol 13, 809-20.

[8] Dornreiter, I., Hoss, A., Arthur, A.K. and Fanning, E. (1990). SV40 T antigen binds directly to the large subunit of purified DNA polymerase alpha. EMBO J 9, 3329-3336.

[9] Weisshart, K., Förster, H., Kremmer, E., Schlott, B., Grosse, F. and Nasheuer, H.P. (2000). Protein-protein interactions of the primase subunits p58 and p48 with simian virus 40 T antigen are required for efficient primer synthesis in a cell-free system. J Biol Chem 275, 17328-17337.

[10] Zhou, B. et al. (2012). Structural basis for the interaction of a hexameric replicative helicase with the regulatory subunit of human DNA polymerase alpha-primase. J Biol Chem 287, 26854-66.
